# Abnormal left atrial compliance is associated with a history of life-threatening arrhythmia in corrected Tetralogy of Fallot

**DOI:** 10.3389/fcvm.2023.1161017

**Published:** 2023-04-26

**Authors:** Mathilde Vautier, Benoit Mulet, Clémence Macquaire, Cynthia Cousergue, Camille-Océane André, Pascale Maragnes, Pierre Ollitrault, Fabien Labombarda

**Affiliations:** ^1^ Department of Cardiology, CHU de Caen-Normandie, Caen, France; ^2^ Department of Pediatrics, CHU de Caen-Normandie, Caen, France; ^3^ Department of Cardiology, Normandie Univ, UNICAEN, CHU Caen-Normandie, UR PSIR 4650, Unicaen, Caen, France

**Keywords:** strain, congenital heart disease, sudden cardiac death, ventricular arrhythmia, compliance

## Abstract

**Objectives:**

The objectives of this study were to examine left atrial (LA) function and compliance using two-dimensional (2D) strain analysis in adult patients with corrected Tetralogy of Fallot (c-ToF) and to investigate the relationships between LA function and patient characteristics, especially history of life-threatening arrhythmia (h-LTA).

**Methods:**

Fifty-one c-ToF patients (34 males; age, 39 ± 15 years; h-LTA, *n* = 13) were included in this retrospective monocenter study. In addition to a 2D standard echocardiography examination, 2D strain analysis was performed to assess left ventricular (LV) and LA functions, including peak-positive LA strain (LAS—reservoir function) and LA compliance [defined as the ratio LAS/(*E*/*Ea*)].

**Results:**

Patients with h-LTA were older and exhibited a longer QRS duration. LV ejection fraction, LAS and LA compliance were significantly lower in the group of patients with h-LTA. Indexed LA and RA volumes, RV end-diastolic area was significantly higher and RV fractional area change significantly lower in the h-LTA group. LA compliance was the best echocardiographic predictor for h-LTA (AUC: 0.839; *p* < 0.001). Moderate inverted correlations were found between LA compliance and age and QRS duration. Among the echocardiographic parameters, LA compliance was moderately inversely correlated with RV end-diastolic area (*r* = −0.40, *p* = 0.01).

**Conclusion:**

We documented abnormal LAS and LA compliance values in adult c-ToF patients. Further study is needed to determine how best to incorporate LA strain, particularly LA compliance, into multiparametric predictive models for LTA in c-ToF patients.

## 1. Introduction

Tetralogy of Fallot (ToF) represents the most common cyanotic heart defect at birth, accounting for 10% of all congenital cardiac defects (1). Although the overall survival of ToF has significantly improved with early surgical repair, long-term cardiac sequelae after corrected ToF (c-ToF), including chronic pulmonary regurgitation, biventricular dysfunction, life-threatening arrhythmias (LTA) and sudden cardiac death, persist (2, 3). The majority of previous studies in c-ToF have mainly focused on the assessment of right and left ventricular function and ventriculo–ventricular interaction with limited attention to atrial function (4). Advances in echocardiographic imaging technology, in particular, 2D strain analysis, have enabled noninvasive evaluation of left atrial (LA) function (5). In recent years, the prognostic utility and clinical relevance of LA function assessed by 2D strain analysis, particularly peak longitudinal LA strain (LAS, reservoir function), has emerged as noteworthy in the prediction of outcomes in different forms of acquired heart disease (6), and there is increasing data regarding its relevance in congenital heart disease (7). Although LA function abnormalities have been previously described in patients with c-ToF (8), LA function remains poorly explored and incompletely characterized late after c-ToF. In addition, its clinical utility has not yet been clearly investigated in this population of patients at risk of premature LV dysfunction and cardiovascular events, particularly cardiac arrhythmia. In this study, we aimed to characterize LA mechanics and function by 2D strain analysis, focusing on LAS (reservoir function) and LA compliance [defined by LAS/(*E*/*Ea*)], in adult patients with c-ToF and to examine the relationships between LA function and patient characteristics, especially history of life-threatening arrhythmia (h-LTA).

## 2. Methods

### 2.1. Population study

**Figure 1:**
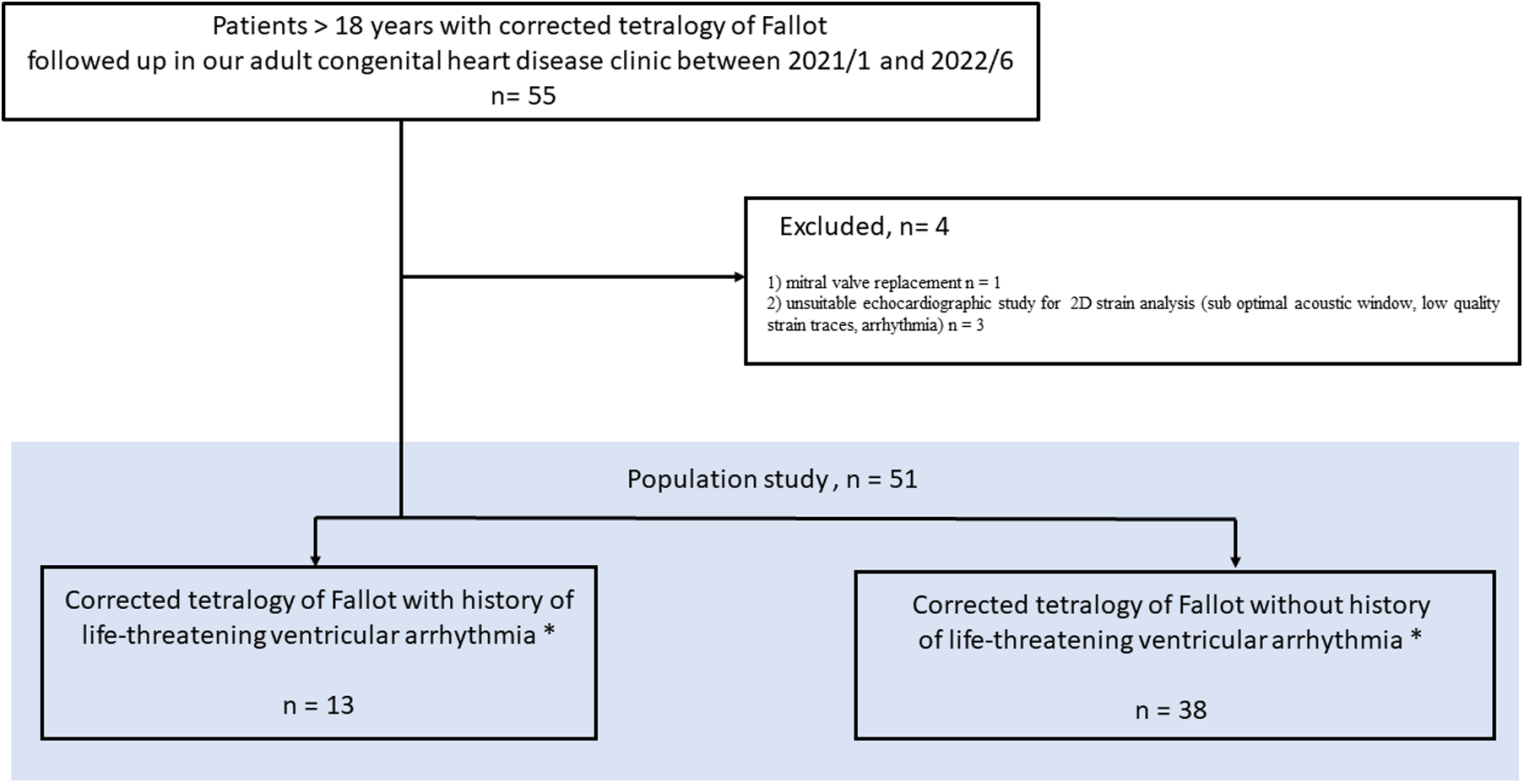
Study flow chart.

Consecutive adult patients with ToF repair who were referred to our outpatient congenital heart clinic from January 2021 to June 2022 for routine echocardiographic follow-up were included in this study. Patient characteristics, including age, sex, QRS duration, medication, history of cardiovascular events and information related to the c-ToF, were collected from medical records and entered in a dedicated database. h-LTA as well as other major cardiovascular events occurring during follow-up after ToF repair (remote from perioperative periods) were listed. h-LTA was defined as a documented episode of sustained ventricular tachycardia (VT) lasting ≥30 s or ventricular fibrillation (VF). Other major cardiovascular events were defined as a history of ischemic stroke, sustained or paroxysmal atrial arrhythmias and heart failure. The flowchart of the patient selection process is presented in Figure 1.

All adult patients with c-ToF were previously registered in our regional database and the CARL initiative (NCT02897323), and the study protocol was approved by our ethical committee.

### 2.2. Echocardiographic assessment

Every subject underwent a 2D trans thoracic echocardiography (TTE) examination (EpiQ 7, X5 probe, Philips® Medical Systems, Andover, MA, USA), including standard echocardiographic views, tissue Doppler imaging (TDI), and 2D strain analysis. The electrocardiogram was recorded continuously. A minimum of 3 cardiac cycles was recorded for each image, and the measurements were averaged accordingly.

Echocardiographic acquisitions were stored on a dedicated workstation for offline analysis using the IntelliSpace Cardiovascular system and QLAb software (Philips® Medical Systems, Andover, MA, USA).

#### 2.2.1. Standard parameters

LV dimensions and LA volumes were measured following the guidelines of both the European Association of Echocardiography and the American Society of Echocardiography (9), and LV filling pressure estimation was performed as well (10). Peak velocities of the E-wave (early diastole) and the A-wave (late diastole) and the E/A ratio were measured from the apical 4-chamber view by pulsed-wave Doppler at the level of the mitral valve tips. Using pulsed-wave TDI, lateral mitral annular peak velocities during early diastole (*Ea*) were obtained, and the mitral *E*/*Ea* ratio was calculated. Right atrium (RA) and right ventricular (RV) conventional measurements were determined according to the guidelines (11). RV measurements included the RV end-diastolic area, fractional area change (FAC), RA volume and tricuspid annular systolic peak velocity by TDI (*S*’). The LV end-diastolic diameter, LA volume and RA volume were normalized to body surface area according to the Dubois formula and expressed in m^2^.

#### 2.2.2. 2D speckle-tracking echocardiography

Myocardial LV and LA strains were analyzed through 2D speckle tracking using aCMQ software (QLAB 10.3; Philips® Medical Systems, Andover, MA, USA). Standard 2D grayscale acquisitions were recorded at a high image rate (>55 Hz) during breath hold and stored for postprocessing analysis. Global longitudinal strain of the LV (LV-GLS) was quantified from the apical 4-, 3- and 2-chamber views (17 segments). Fast, semiautomatic contouring of the endocardium was achieved by placing three points (basal septum, basal lateral wall, and apex) at the endocardium. The software then suggested a region of interest of adjustable thickness that could be repositioned by the operator. When myocardial tracking was considered optimal, the software analyzed the global and segmental strains and represented them as colored curves. LV-GLS was obtained by averaging all segmental values. Peak positive longitudinal LA strain (LAS, reservoir function) was assessed from the apical four-chamber view by semiautomatically tracing and tracking the entire endocardial contour of the LA. Strain values were derived from the time‒strain, averaging 6 atrial segments and taking the QRS complex as the reference point. Finally, LA compliance was estimated by taking the quotient of LAS reservoir strain and *E*/*Ea* as previously proposed (12).

### 2.3. Statistical analysis

A population study was described, and a comparative analysis of patients with ToF repair with h-LTA and those without h-LTA was performed. Continuous variables are presented as the mean ± SD or median as appropriate. Categorical variables were expressed as frequencies and percentages. Between-group differences were analyzed using Student's *t* test, Mann–Whitney test, *χ*^2^ or Fisher's exact tests as appropriate. For all parameters with a univariate *p* < 0.05, a receiver operator characteristic (ROC) curve was plotted with the corresponding area under the curve (AUC) calculations to assess the ability to discriminate ToF patients with h-LTA. Correlations were tested using Pearson coefficients. Intraclass correlation coefficients (ICCs) and their 95% confidence intervals were calculated in a randomly chosen sample of 15 participants to assess the reliability of the LAS and LA compliance measurements. Statistical significance was defined as *p* < 0.05. Data were analyzed using MedCalc version 18.11.6.

## 3. Results

### 3.1. Population study

A total of 51 patients (66.5% male, age 39 ± 15 years) with c-ToF were enrolled in this monocenter study. A flow chart is depicted in Figure 1. The characteristics of the study population are summarized in Table 1. The surgical repair of ToF had been performed at a mean age of 4.6 years, 22 (43%) patients had a history of prior palliative shunt, and 20 (39%) had pulmonary valve replacement. One patient had a liver cirrhosis caused by alcohol consumption; 2 patients had moderate to severe renal dysfunction in h-LTA group while no patient had renal dysfunction in group without h-LTA. Thirteen patients had h-LTA (25.5%), of whom 12 benefited from transcatheter VT ablation, and 11 were implanted with ICD (21.5%). Two patients refused ICD implantation. Finally, 16 patients presented with atrial arrhythmia (31.5%). Ventricular and atrial arrhythmia are depicted in Table 2.

**Table 1:** Baseline characteristics of the study population.

Variables	cToF population (*n* = 51)	cToF patients with h-LTA (*n* = 13)	cToF patients without h-LTA (*n* = 38)
Age (years)	39 ± 15	49 ± 8	35 ± 14
Gender, male (%)	34 (66.5%)	10 (77%)	23 (62%)
QRS duration on EKG (ms)	141 ± 29	160 ± 27	135 ± 28
NYHA I/II/III/IV	40/7/4	8/4/1/0	32/3/3/0
Syncope	2 (3.9%)	1 (7.7%)	1 (2.7%)
Hypertension	5 (9.8%)	2 (15.4%)	3 (8.1%)
Diabetes	1 (1.9%)	1 (7.7%)	0
Current smoking history	5 (9.8%)	2 (15.4%)	3 (8.1%)
Creatinine (micromol/L)	82 ± 34	101 ± 51	73 ± 16
ASAT/ALAT^a^ (U/L)	32 ± 15	29 ± 10	31 ± 12
Medication			
ACE inhibitor/angiotensin II antagonist	5 (9.8%)	1 (7.7%)	4 (10.8%)
Beta blocker	18 (35%)	11 (84.6%)	7 (19%)
Diuretics	10 (19.5%)	5 (38.5%)	5 (13.5%)
Anti-arrhythmic drugs amiodarone/others	5 (9.8%)	3 (23%)	2 (5.4%)
Aspirin	5 (9.8%)	3 (23%)	2 (5.4%)
Anticoagulation	13 (25.5%)	6 (46.2%)	7 (19%)
Tetralogy of Fallot history			
Prior palliative shunt	22 (43%)	6 (42.5%)	18 (47.5%)
Age at corrective surgery (years)	4.6 (0.3–18)	7.4 ± 5.5	4.7 ± 3.7
Pulmonary valve replacement	20 (39%)	11 (78.5%)	13 (35%)
Pacemaker/Defibrillator	11 (21.5%)	11 (84.6%)	0
History of Cardiovascular events			
Life-threatening arrhythmia^b^	13 (25.5%)	13	0
Sudden cardiac death	0	0	0
Atrial arrhythmia^c^	16 (31.5%)	7 (54%)	9 (23.5%)
Stroke	0	0	0
Heart failure	7 (13.5%)	4 (30.8%)	3 (8%)

cToF, corrected Tetralogy of Fallot; NYHA, New York heart association.

^a^ Referent value ASAT [5–35 U/L], ALAT [5–45 U/L].

^b^ Life threatening arrhythmia included documented sustained ventricular arrhythmia and ventricular fibrillation.

^c^ Atrial arrhythmia included atrial fibrillation, intra atrial reentrant tachycardia and and focal atrial tachycardia.

**Table 2:** Arrhythmia burden in patients with repaired Tetralogy of Fallot.

Characteristics	Study population (*n* = 51)	cToF patients with h-LTA (*n* = 13)	cToF patients without h-LTA (*n* = 38)
Ventricular tachyarrhythmia			
VT	13 (25.5%)	13 (100%)	0
VF	0	0	0
Atrial tachyarrhythmia	16 (31.5%)	7 (54%)	9 (23.5%)
AF	8 (15.5%)	5 (38.5%)	3 (8%)
IART	8 (15.5%)	2 (15.5%)	6 (15.5%)
FAT	1 (1.9%)	1 (7.5%)	0
Transcatheter VT ablation	12 (23.5%)	12 (92%)	0
ICD	11 (21.5%)	11 (84.5%)	0

VT, ventricular tachycardia; VF, ventricular fibrillation; AF, atrial fibrillation; IART, intra atrial reentrant tachycardia; FAT, focal atrial tachyarrhythmia; ICD, intra cardiac defibrillation (one patient may have more than one type of arrhythmia).

### 3.2. Patients with or without h-LTA

**Figure 2:**
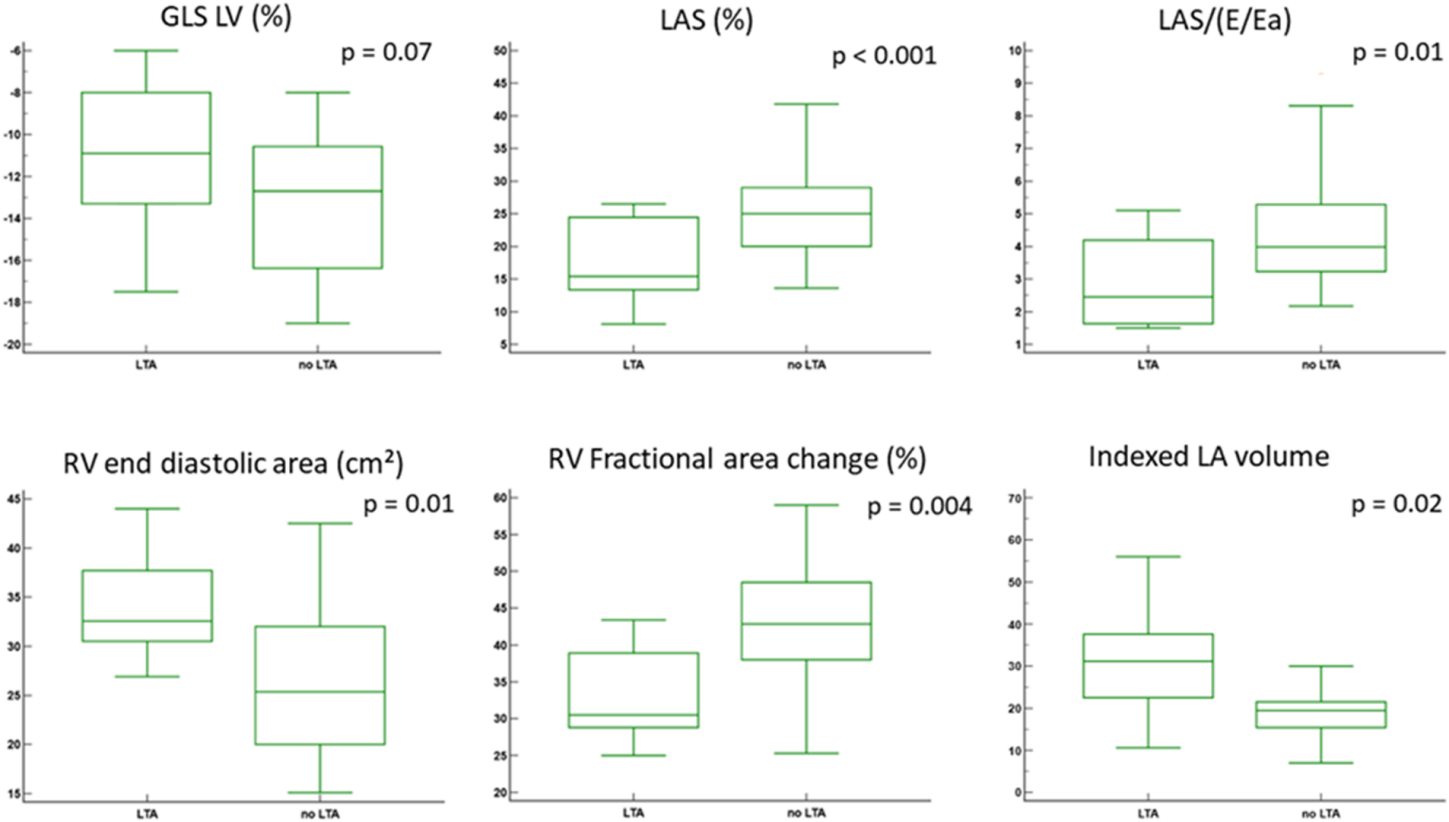
Comparisons of echocardiographic parameters according to h-LTA. GLS, global longitunal strain; LV, left ventricle; RV, right ventricle; LAS, left atrial strain.

The results are presented in Table 3 and Figure 2. All 13 of the 51 ToF patients with h-LTA (29%) presented sustained VT. Compared to patients without h-LTA, these participants were older, exhibited a longer QRS duration and were associated with a greater incidence of pulmonary valve replacement. Age at corrective surgery was higher in the h-LTA group, but the difference did not reach statistical significance. LV ejection fraction, LAS and LA compliance were significantly lower in the group of patients with h-LTA, whereas LV dimensions and LV filling parameters did not differ. Indexed LA and RA volumes, RV end-diastolic area was significantly higher and RV fractional area change significantly lower in the h-LTA group. LV-GLS was lower in this group, but the difference was not statistically significant.

**Table 3:** Characteristics of patients with repaired Tetralogy of Fallot (ToF) according to life threatening arrhythmia (LTA).

Variables	ToF with LTA history (*n* = 13)	ToF without LTA history (*n* = 38)	*p*
Age (years)	49 ± 8	35 ± 14	**<0.001**
Gender, Male (%)	10 (77%)	23 (62%)	0.6
QRS duration (ms)	160 ± 27	135 ± 28	**<0**.**001**
Prior palliative shunt	6 (42.5%)	18 (47.5%)	0.06
Age at corrective surgery (years)	7.4 ± 5.5	4.7 ± 3.7	0.06
Pulmonary valve replacement	11 (78.5%)	13 (35%)	**0**.**03**
Standard Echocardiographic measurements			
LV ejection fraction (%)	56 ± 12	63 ± 7	**0**.**03**
Indexed LV end diastolic diameter (mm/m^2^)	34 ± 13	32 ± 8	0.41
Mitral *E*/*A* ratio	1.4 ± 0.3	1.6 ± 0.7	0.16
Lateral *Ea* mitral annular velocity by TDI (cm/s)	12.8 ± 2.1	13.4 ± 3	0.26
Mitral *E*/*Ea* ratio	6.7 ± 2.1	6.1 ± 2.8	0.09
Indexed left atrial volume (ml/m^2^)	31 ± 15	24 ± 10	**0**.**02**
RV end diastolic area (cm^2^)	33 ± 5	26 ± 7	**0**.**01**
RV Fractional area change (%)	34 ± 6	43 ± 8	**0**.**004**
Tricuspid annular systolic velocity by DTI (cm/s)	9.1 ± 2	9.8 ± 2.4	0.12
Indexed RA volume (ml/m^2^)	44 ± 30	33 ± 16	**0**.**001**
Strain measurements			
LV-GLS (%)	−11 ± 3	−13.2 ± 3	0.07
LAS (%)	17 ± 6	26 ± 7	**<0**.**001**
LA compliance			
LAS/(*E*/*Ea*)	3 ± 1.8	4.5 ± 1.9	**0**.**01**

LV, left ventricle; RV, right ventricle; TDI, tissue Doppler imaging; RA, right atrial; LA, left atrial; GLS, global longitudinal strain; LAS, peak positive left atrial strain; LTA, life threatening arrhythmia, including documented sustained ventricular arrhythmia and ventricular fibrillation.

Bold value represents statistically significant difference.

### 3.3. Potential ability of echocardiographic measurements to predict h-LTA in c-TOF patients

**Figure 3:**
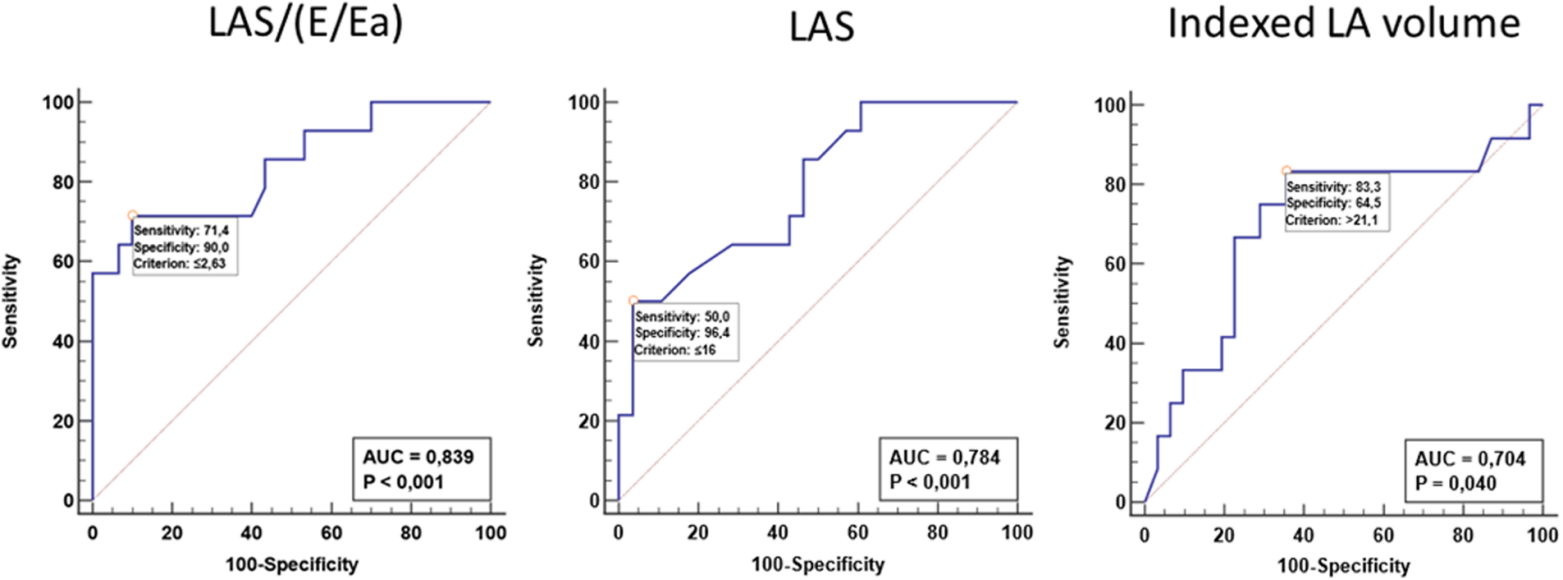
Receiver operating characteristic curves receiver operator characteristic (ROC) curve with the corresponding area under the curve (AUC) calculations to assess the ability to discriminate ToF patients with h-LTA. LA, left atrium; LAS, left atrial strain (reservoir function).

**Figure 4:**
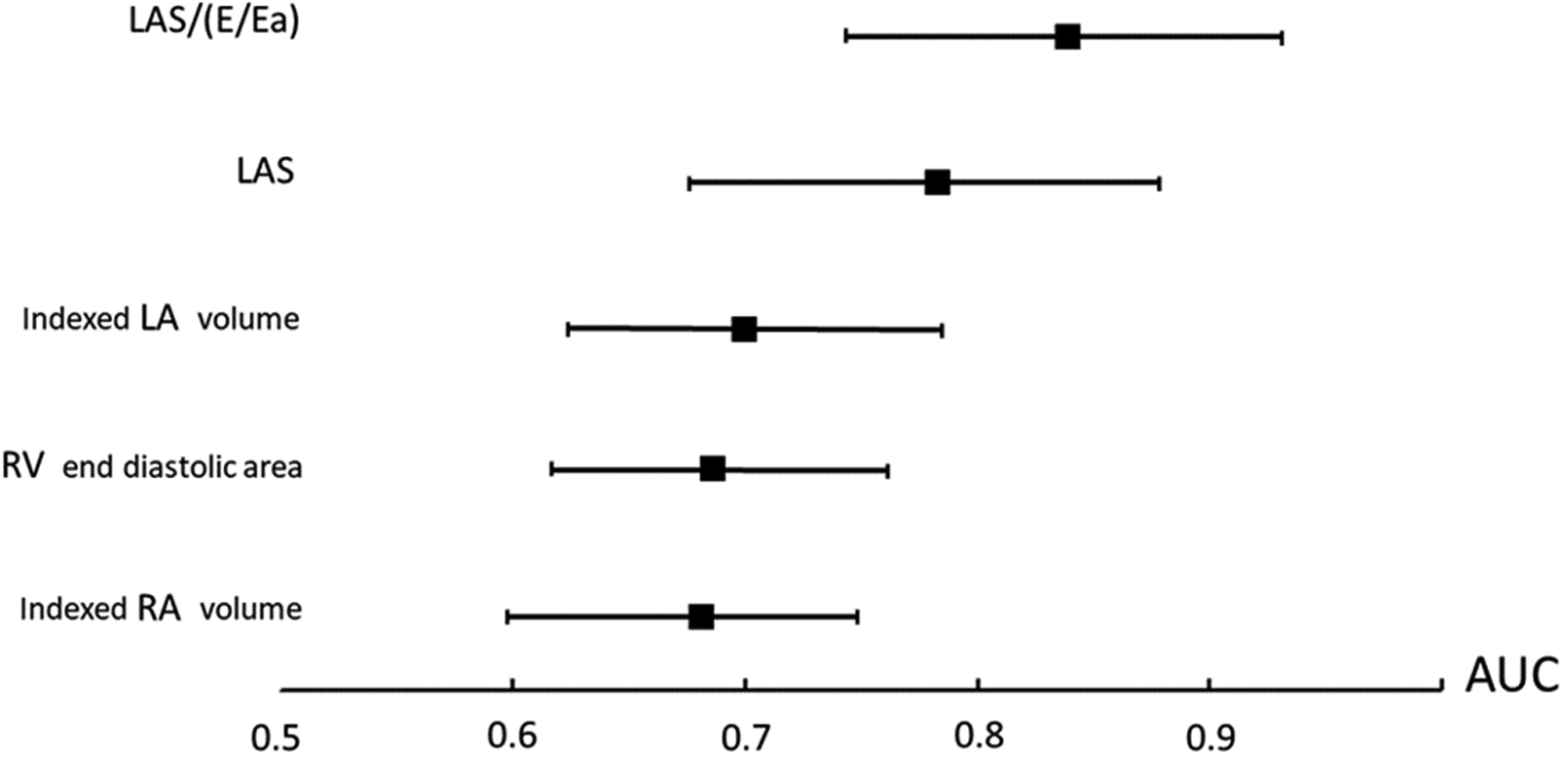
Comparisons of area under the curve. LA, left atrium; LAS, left atrial strain (reservoir function); Ra, right atrium; RV, right ventricle.

Among echocardiographic parameters, LA compliance was the best predictor of h-LTA (AUC: 0.839; *p* < 0.001, Figures 3, 4). The diagnostic performance of LA compliance, LAS and indexed LA volume, along with the proposed cutoff values, are presented as receiver operating characteristic curves (Figure 3).

### 3.4. Relationships between LA compliance strain and clinical/echocardiographic parameters

Moderate inverted correlations were found between LA compliance and age and QRS duration. Among the echocardiographic parameters, LA compliance was moderately inversely correlated with RV end-diastolic area (*r *= −0.40, *p *= 0.01). No significant associations were found among the remaining echocardiographic parameters. The associations among LA compliance, patient characteristics, and echocardiographic measurements are reported in Table 4.

**Table 4:** Correlations with LA compliance^a^.

	Correlation coefficient	*p*
Patients characteristics		
Age (years)	−0.38	**0**.**017**
QRS duration (ms)	−0.31	**0**.**04**
Echocardiographic measurements		
LV ejection fraction (%)	0.21	0.1
Indexed left atrial volume (ml/m^2^)	−0.3	0.07
Indexed RA volume (ml/m^2^)	−0.32	0.05
RV end diastolic area (cm^2^)	−0.18	0.17
RV fractional area change (%)	−0.40	**0**.**03**
GLS strain (%)	−0.3	0.05

LV, left ventricle; RV, right ventricle; GLS, global longitudinal strain; LAS, pak positive left atrial strain (%).

Bold value represents statistically significant difference.

^a^ Defined by LAS/(*E*/*Ea*).

### 3.5. Interobserver reproducibility and intraobserver repeatability

Analysis of intraobserver and interobserver variability demonstrated good agreement between observations for the LAS [ICC (95% confidence interval) 0.94 (0.84–0.99) and 0.87 (0.62–0.97), respectively] as well as for the LA compliance [ICC (95% confidence interval): 0.96 (0.84–0.99) and 0.88 (0.64–0.97), respectively].

## 4. Discussion

In our cohort of adult patients with c-ToF, we found alterations in LA function with a marked decrease in LAS and LA compliance in the group of patients with h-LTA. Among echocardiographic parameters, LA compliance was the best predictor of h-LTA. Although preliminary, our findings suggest a potential benefit of combining LA compliance analysis in c-ToF monitoring to identify a subgroup of patients at risk of LTA.

### 4.1. Left atrial mechanics and function after ToF repair

Unlike RV function, LA function long after ToF surgery has been addressed in few studies, although the clinical and prognostic relevance of LA function are increasingly recognized as correlates of outcome (13). The impairment of LAS (reservoir function) observed in our study is in concordance with previous reports using different techniques for LA strain quantification, including TTE 2D/3D speckle strain analysis or MRI (14–16). Several factors may play a role in the impairment of LA reservoir function in c-TOF, such as changes in LV function. Mildly reduced LV function is not uncommon in c-ToF (17). Shared myocardial fibers between the LV and RV, the adverse impact of septal shift due to the effects of pulmonary regurgitation on RV volume overload coupled with electrical dyssynchrony from a bundle branch block (18) and the severity of preoperative hypoxemia (19) have been suggested to account for LV dysfunction, in particular, LV longitudinal function (20). Thus, these mechanisms may impact LA reservoir function, which is mainly modulated by LV longitudinal function through the downward motion of the mitral plane during ventricular systole. The potential interactions between both RA and LA (15), including interatrial transmission of hemodynamic changes across the atrial septum (21), may also explain the LAS and LA compliance changes in c-ToF patients. Finally, RV dilation and dysfunction, a classical long-term feature after c-ToF surgery because of ventriculotomy, transannular patch and chronic pulmonic and/or tricuspid regurgitation (22), may be responsible for RA dilation and dysfunction (23), indirectly impairing LA function. The correlations between LA compliance and RV end-diastolic area as well as QRS widening identified in our study are in keeping with these possible physiopathological mechanisms. We found an apparent unexpected behaviour of *S*’ compared to FAC. Lack of sensitivity of commonly used *S*’ and tricuspid annular plane systolic excursion as markers for global RV function was previously reported in both adults and children with c-TOF (24, 25). In the study of Selly et al., *S*’ showed no significant correlation with global systolic RV function using MRI while FAC was significantly correlated with RV ejection fraction estimated by MRI (26). The altered regional contraction pattern associated with the abnormal RV shape and the importance of the RV apex remodeling compared to the base, were proposed as potential mechanisms for the lack of sensitivity of longitudinal shortening variables in patients with c-TOF (26). These previous reports may explain our results regarding FAC and *S*’ in our two groups of c-TOF patients.

### 4.2. Left atrial function and life-threatening ventricular arrhythmia after ToF repair

In our study, LAS and LA compliance were significantly decreased in the group of c-ToF patients with h-LTA. The important role of LV function in directing outcomes in c-ToF patients is increasingly recognized. LA strain provides an early marker of LV diastolic and systolic dysfunction. The association between LV systolic/diastolic dysfunction and LTA in c-ToF was previously suggested (27). In a large monocenter study conducted by Diller et al., LV longitudinal function predicted LTA and death in adults with c-ToF (20). In addition, in a multicenter study, Khairy et al. identified elevated LV end-diastolic pressure measured invasively (>12 mm Hg) as the most powerful predictor of appropriate shocks in intracardiac defibrillator recipients with c-ToF (28). The prognostic value of an increased LV end-diastolic pressure was also reported in 3,024 consecutive patients undergoing cardiac surgery (29). Reduced LAS was found to be strongly correlated with LV diastolic function and invasively assessed LV end-diastolic pressures (30). Our results are consistent with these previous reports, which clearly showed the relationship between diastolic LV dysfunction, increased LV end-diastolic pressure and severe ventricular arrhythmias in c-ToF patients. Reasons why LV diastolic dysfunction is strongly associated with ventricular arrhythmias remain speculative. It may be that LV diastolic dysfunction reflects, in part, LV systolic dysfunction, which has previously been linked to sudden death in c-ToF patients (31) as well as to comorbidities such as diabetes mellitus, hypertension, hypercholesterolemia, and obesity. Diastolic dysfunction has been independently associated with ventricular arrhythmias and mortality in other diseases, such as chronic renal failure, sickle cell anemia, and postcoronary bypass surgery. The majority of previous studies on LA strain analysis have focused on LAS (reservoir function), which provides incremental prognostic information in general and referral populations (32, 33). Recently combined echocardiographic parameters, including LA compliance and LA stifness, were proposed for the evaluation of LV diastolic function and filling pressure (12, 34) LA compliance refers to the ability of the LA to stretch and expand in response to an increase in blood volume. It is an important physiological property of the LA that allows it to accommodate blood returning from the lungs and to maintain adequate cardiac output. Indexing LAS reservoir strain to estimated LA pressure (*E*/*E*′) was proposed as a surrogate for LA compliance and was shown to outperform LAS as well as other commonly used echocardiographic criteria used in the evaluation of diastolic dysfunction and high filling pressure (12). In concordance with these findings, we found that LA compliance was the best predictor of h-LTA. Normalizing LA strain to estimated filling pressures (*E*/*e*′) may be more acurate to detect elevated filling pressure in the specific population of cTOF in whom standard parameters were reported to be less performant compared to general population (35). Our findings are in keeping with previous studies having demonstrated the potential interest of another combined echocardiographic parameters including LA stiffness estimated with the ratio (*E*/*E*′)/LAS (36, 37). LA stiffness was shown to be accurate in providing important information on LA function as well as a prognostic factor (34).

### 4.3. Clinical implications

Although rare, malignant ventricular arrhythmias and sudden cardiac death may occur late in c-ToF. Numerous factors have been proposed to identify patients at risk of LTA; nevertheless, identifying these patients in advance is still challenging. As previously mentioned, there is a growing body of evidence on the importance of precisely evaluating LV function and LV filling pressures in c-ToF. Egbe et al. found indexed LA volume to have a moderate discriminatory ability to dectect LV elevated pressure and demonstrated that most of the echocardiographic indices endorsed by the practice guidelines (35) had very poor performance in the assessment of left heart filling pressures in the c-TOF population. The use of other echocardiographic indices for evaluating LV filling pressure therefore appears necessary in this specific population. LA strain analysis is a noninvasive means of assessing LV filling pressure and is now an essential complement in the analysis of LV function. Our results support the argument for the use of LAS and LA compliance in the monitoring of patients after c-ToF. Although preliminary, our findings suggest the potential benefit of combining LA compliance analysis in c-ToF to identify a subgroup of patients at high risk for LTA who may benefit from closer monitoring or specific strategies such as implantable cardiac defibrillators.

### 4.4. Study limitations

Several limitations to this study should be addressed. First, our data must be regarded as preliminary due to the monocenter retrospective study design as well as the relatively small

population resulting from the strict exclusion criteria and the inherent problem with rare diseases. Second, we could not completely exclude the effects of cardiovascular risk factors on LAS and LA compliance values, as well as the presence of subclinical coronary artery disease, which might have impacted LA function. Third, we did not provide a global longitudinal LA strain analysis, with three apical cavities, as previously proposed. However, the meta-analysis conducted by Pathan et al. (38) of studies using a four-chamber view only, four- and two-chamber views, and four-, two-, and three-chamber views provided similar results with no important difference.

## 5. Conclusion

We documented abnormal LAS and LA compliance values in adults after ToF repair. LA compliance allowed for discrimination of h-LTA with greater accuracy compared to other tested echocardiographic indices. Further prospective study is indicated to validate these results and establish a definitive cutoff for abnormal LA compliance values for application in a multiparametric predictive model of LTA in c-ToF.

## Data Availability

The raw data supporting the conclusions of this article will be made available by the authors, without undue reservation.
